# Arbuscular Mycorrhizal Fungi Modulate the Temporal Defense Response of *Passiflora edulis* to *Fusarium oxysporum*

**DOI:** 10.3390/ijms27219495

**Published:** 2026-10-24

**Authors:** Brunara Evely de Araujo Lima, Luiz Victor de Almeida Dantas, Roberta Lane de Oliveira Silva, Natoniel Franklin de Melo, Adriana Mayumi Yano-Melo

**Affiliations:** 1 Programa de Pós-Graduação em Agronomia-Produção Vegetal, Campus Ciências Agrárias, Universidade Federal do Vale do São Francisco, Petrolina CEP 56300-990, PE, Brazil; 2 Programa de Pós-Graduação em Recursos Genéticos Vegetais, Universidade Estadual de Feira de Santana, Feira de Santana CEP 44036-900, BA, Brazil; 3 Unidade Divinópolis, Universidade do Estado de Minas Gerais, Divinópolis CEP 35501-170, MG, Brazil; 4 Embrapa Semiárido, Petrolina CEP 56302-970, PE, Brazil; 5 Laboratório de Microbiologia, Campus Ciências Agrárias, Universidade Federal do Vale do São Francisco, Petrolina CEP 56300-990, PE, Brazil

**Keywords:** arbuscular mycorrhizal fungi, biotic stress, fusaric acid, Fusarium wilt, gene expression, passion fruit, plant defense, qPCR

## Abstract

Arbuscular mycorrhizal fungi (AMF) can modulate plant responses to soil-borne pathogens, but the molecular mechanisms underlying this interaction remain poorly understood in passion fruit. This study investigated the temporal defense response of *Passiflora edulis* inoculated with the AMF *Gigaspora albida* and *Entrophospora etunicata* and subsequently challenged with *Fusarium oxysporum* f. sp. *passiflorae* (FoP) and/or fusaric acid (FA). Plant growth, mycorrhizal colonization, and the expression of five defense- and signaling-related genes (*PePR-1*, *PePR-12*, *PeSERK1*, *PeLecRK*, and *PeWRKY*) were evaluated. AMF inoculation significantly enhanced vegetative growth, increasing plant height, leaf number, and stem diameter. Gene expression showed a marked time-dependent response. At 24 h after stress induction, most candidate genes were downregulated, whereas after seven days *PePR-12*, *PeSERK1*, *PeLecRK*, and *PeWRKY* were induced in most treatments. Notably, *PeWRKY* was induced in all treatments at seven days and showed particularly high expression in FoP-challenged plants. Mycorrhizal and arbuscular intensities were strongly positively correlated with *PePR-12* expression at 24 h, while at seven days *PeLecRK* expression was strongly correlated with *PeSERK1*, *PeWRKY*, and *PePR-12*. These results reveal a dynamic temporal reprogramming of defense-related gene expression in *P. edulis* and indicate that AMF colonization is associated with modulation of molecular responses to FoP. The identified genes provide candidates for further functional investigation of mycorrhiza-mediated defense responses in passion fruit.

## 1. Introduction

Passion fruit (*Passiflora edulis* Sims) is an economically important tropical fruit crop, with Brazil being one of its major producers. The fruits are widely consumed fresh and processed into juices and other products, making the crop particularly relevant to tropical and subtropical fruit production systems. However, the establishment, productivity, and longevity of commercial orchards are strongly constrained by soil-borne diseases, among which Fusarium wilt is particularly destructive [1,2].

Fusarium wilt of passion fruit is associated with *Fusarium oxysporum* f. sp. *passiflorae* (FoP), a soil-borne vascular pathogen capable of causing severe losses in susceptible plants. Infection is associated with vascular colonization and dysfunction, followed by leaf yellowing, progressive wilting, root and shoot decline, and ultimately plant death [2,3]. Disease management is challenging because the pathogen can persist in infested soil and effective curative control is lacking. Moreover, commercial *P. edulis* materials are generally susceptible, and considerable effort has therefore been directed toward identifying resistant *Passiflora* genotypes and rootstocks [2,3].

In addition to fungal colonization itself, secondary metabolites produced by *Fusarium* spp. can contribute to disease development. Fusaric acid (FA) is a non-host-specific phytotoxic metabolite produced by several *Fusarium* species and has been associated with oxidative stress, disruption of membrane functions, altered water and mineral homeostasis, and cellular damage in plants [4,5]. Importantly, genetic disruption of FA biosynthesis reduces the virulence of *F. oxysporum*, providing direct evidence that this metabolite contributes to the infection process [4]. Recent comparative transcriptomic, chemical, and genetic analyses have further supported a role for FA as a positive virulence factor during *F. oxysporum* infection [5]. Therefore, comparing plant responses to FoP with those elicited by FA provides an experimental framework for distinguishing responses to the living pathogen from those triggered by an important component of its phytotoxic arsenal.

Arbuscular mycorrhizal fungi (AMF) establish widespread mutualistic associations with plant roots and can improve mineral nutrition, water acquisition, growth, and tolerance to environmental stresses [6,7]. Beyond these effects, AMF can profoundly modify host signaling and defensive capacity. This phenomenon, commonly referred to as mycorrhiza-induced resistance (MIR), can enhance the defensive state of mycorrhizal plants against diverse attackers and shares mechanistic features with other forms of induced resistance [8]. Establishment of AM symbiosis itself involves extensive molecular communication between the fungal and plant partners, including signaling molecules, membrane receptors, calcium signaling, transcription factors, and regulatory RNAs, ultimately resulting in extensive transcriptional reprogramming of the host [6]. Transcriptomic analyses have also shown that AMF colonization can alter genes associated with cell-wall remodeling, membrane functions, transport, metabolism, and defense [7]. Thus, AMF-associated protection should not necessarily be interpreted as constitutive activation of plant defense, but rather as a reconfiguration of the host signaling state that may modify the timing and magnitude of subsequent responses to stress.

At the molecular level, this response involves multiple regulatory layers, from extracellular signal perception to transcriptional regulation and downstream defense responses. Somatic embryogenesis receptor-like kinases (SERKs) function as coreceptors in receptor complexes and participate in signaling processes associated with plant development and immunity [9,10]. Lectin receptor-like kinases (LecRKs), in turn, are plasma membrane-associated receptors capable of perceiving extracellular signals and participating in the activation and regulation of innate immune responses [11]. Downstream of receptor-mediated signaling, WRKY transcription factors constitute an important regulatory hub connecting stress perception to transcriptional reprogramming and defense-related responses [12]. Pathogenesis-related (PR) proteins represent another important component of the plant immune response and are frequently used as molecular indicators of defense activation [13].

Genomic and transcriptomic resources developed for *P. edulis* have begun to reveal the complexity of its stress-responsive regulatory networks. Genome-wide studies have identified members of several transcription-factor and defense-related gene families whose expression is altered by biotic or abiotic stress. NAC genes, for example, exhibit differential expression following *Fusarium* challenge and drought stress [14], while bHLH genes show extensive stress-responsive expression patterns [15]. Members of the KCS gene family also respond to *Fusarium* and drought treatments [16], and genome-wide characterization of the WRKY family has identified *PeWRKY* genes potentially involved in stress-responsive signaling in passion fruit [17]. Collectively, these studies demonstrate that *P. edulis* possesses a complex and dynamic molecular response to environmental challenges. However, previous studies have largely focused on individual gene families or on the direct response of passion fruit to stress, whereas the molecular interface between a beneficial AM symbiosis and the defense response to Fusarium wilt remains poorly understood.

In particular, it remains unclear whether prior AMF colonization alters the temporal coordination of pathogen perception, signal transduction, transcriptional regulation, and downstream defense responses in *P. edulis* challenged by FoP. Moreover, the extent to which this modulation differs between the response to the living pathogen and that elicited by FA has not been characterized. Addressing this gap is particularly relevant because AMF-mediated effects may involve changes in the kinetics of defense activation rather than a simple constitutive increase in defense-gene expression.

We therefore hypothesized that prior AMF colonization reprograms the temporal defense response of *P. edulis* to FoP and FA. To test this hypothesis, plants colonized by *Gigaspora albida* and *Entrophospora etunicata* were challenged with FoP, FA, or their combination, and responses were evaluated at two contrasting time points, 24 h and seven days after challenge. Plant growth and root mycorrhizal colonization were assessed together with the expression of genes representing different levels of the defense network: *PeSERK1* and *PeLecRK*, associated with receptor-mediated signaling; *PeWRKY*, associated with transcriptional regulation; and *PePR-1* and *PePR-12*, associated with downstream defense responses. By integrating prior AMF colonization, pathogen and phytotoxin challenges, and temporally resolved expression of genes acting at different levels of the defense network, this study provides evidence that the AMF-associated response of passion fruit is characterized by temporal reprogramming rather than by a simple constitutive enhancement of defense-gene expression.

## 2. Results

### 2.1. AMF Inoculation Enhances Vegetative Growth of P. edulis

AMF inoculation significantly enhanced the vegetative growth of *P. edulis* seedlings (Table 1). Plant height was significantly greater in AMF-inoculated than in non-inoculated plants at 30, 45, and 70 days after inoculation (F = 49.582, *p* < 0.001). At 30 days, AMF-inoculated plants reached an average height of 15.47 cm compared with 11.69 cm in non-inoculated plants. At 45 days, mean plant height was 43.33 cm in AMF-inoculated plants and 19.49 cm in non-inoculated plants, whereas at 70 days these values were 89.92 and 61.12 cm, respectively.

Leaf number (F = 20.442, *p* < 0.001) and stem diameter (F = 46.732, *p* < 0.001) did not differ significantly between AMF-inoculated and non-inoculated plants at 30 days, but significant differences were detected at both 45 and 70 days. At 45 days, AMF-inoculated plants had an average of 13.08 leaves and a stem diameter of 0.41 cm, compared with 10.05 leaves and 0.14 cm in non-inoculated plants. At 70 days, AMF-inoculated plants had 14.16 leaves and a stem diameter of 0.59 cm, whereas non-inoculated plants had 12.47 leaves and a stem diameter of 0.48 cm (Table 1).

Overall, AMF-inoculated plants exhibited greater vegetative growth than non-inoculated plants, with significant effects on plant height from 30 days onward and on leaf number and stem diameter from 45 days onward.

### 2.2. AMF Inoculation Results in Greater Root Colonization and Arbuscule Formation

**Figure 1:**
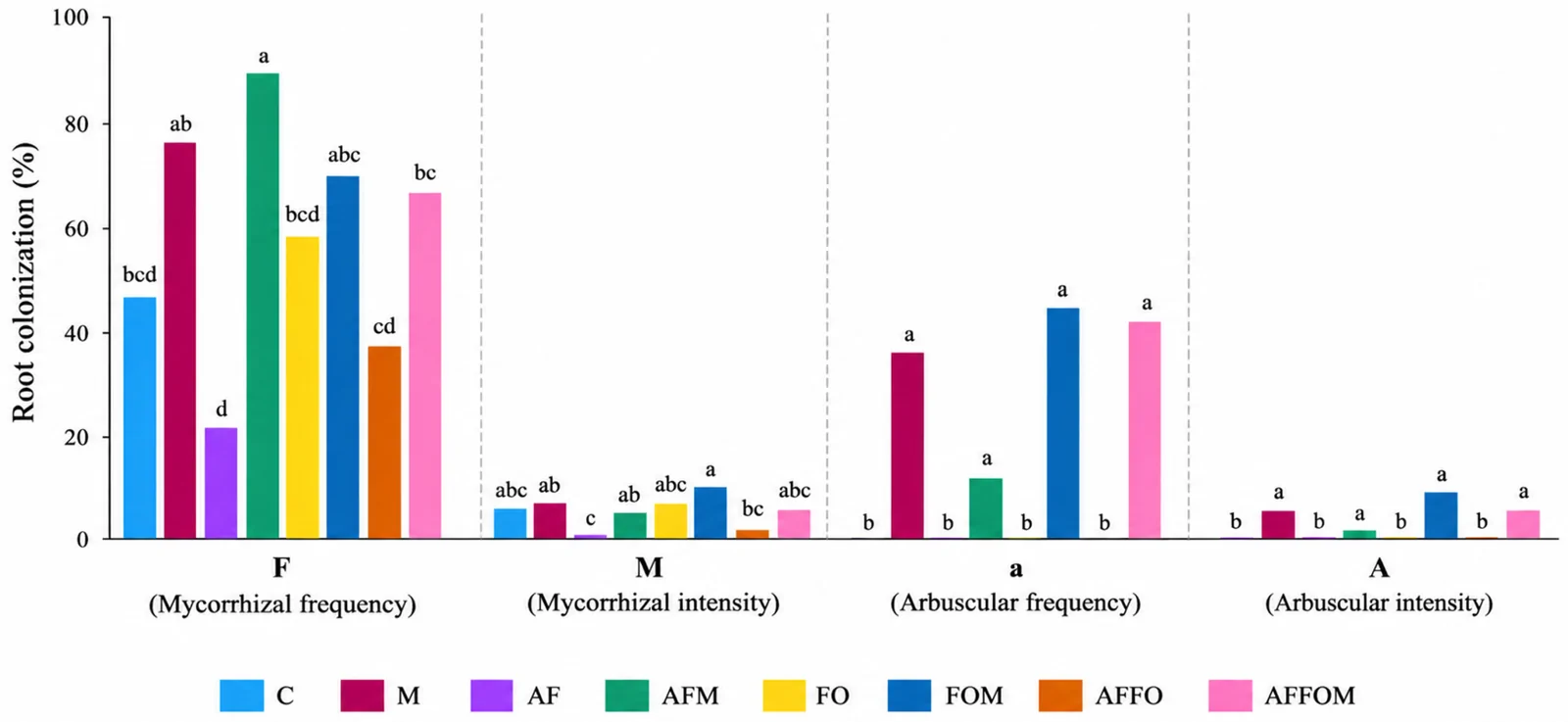
Mycorrhizal colonization and arbuscule formation in roots of *Passiflora edulis* at the end of the experiment. Mycorrhizal frequency (F), mycorrhizal intensity (M), arbuscular frequency (a), and arbuscular intensity (A) were determined according to Trouvelot et al. [18]. Treatments: C = non-inoculated control; M = AMF-inoculated; AF = fusaric acid; AFM = AMF-inoculated + fusaric acid; FO = *Fusarium oxysporum* f. sp. *passiflorae* (FoP); FOM = AMF-inoculated + FoP; AFFO = fusaric acid + FoP; AFFOM = AMF-inoculated + fusaric acid + FoP. Different lowercase letters above the bars indicate significant differences among treatments for each colonization variable according to Tukey’s test (*p* < 0.05).

Root colonization assessed at the end of the experiment varied among treatments (Figure 1). Mycorrhizal frequency (F) was highest in the AFM treatment, whereas lower values were observed in several non-AMF-inoculated treatments. Mycorrhizal intensity (M) also varied among treatments. Fungal colonization was also detected in treatments that had not been intentionally inoculated with AMF. In these treatments, mycorrhizal frequency (F) ranged from 19.17% to 57.50%, while mycorrhizal intensity (M) ranged from 0.26% to 5.71%, considering both evaluation times. Importantly, no arbuscules were detected in any of the non-inoculated treatments, with both arbuscular frequency (a) and arbuscular intensity (A) remaining at 0%. These findings indicate that, despite the presence of fungal structures, there was no morphological evidence of an established functional mycorrhizal association in non-inoculated plants. Arbuscule-related variables more clearly distinguished AMF-inoculated from non-inoculated plants. Arbuscular frequency (a) exceeded 10% only in treatments previously inoculated with AMF. No significant differences in arbuscular frequency or intensity were detected among the AMF-inoculated treatments (Figure 1).

### 2.3. Defense-Related Gene Expression Shows a Pronounced Temporal Shift

**Figure 2:**
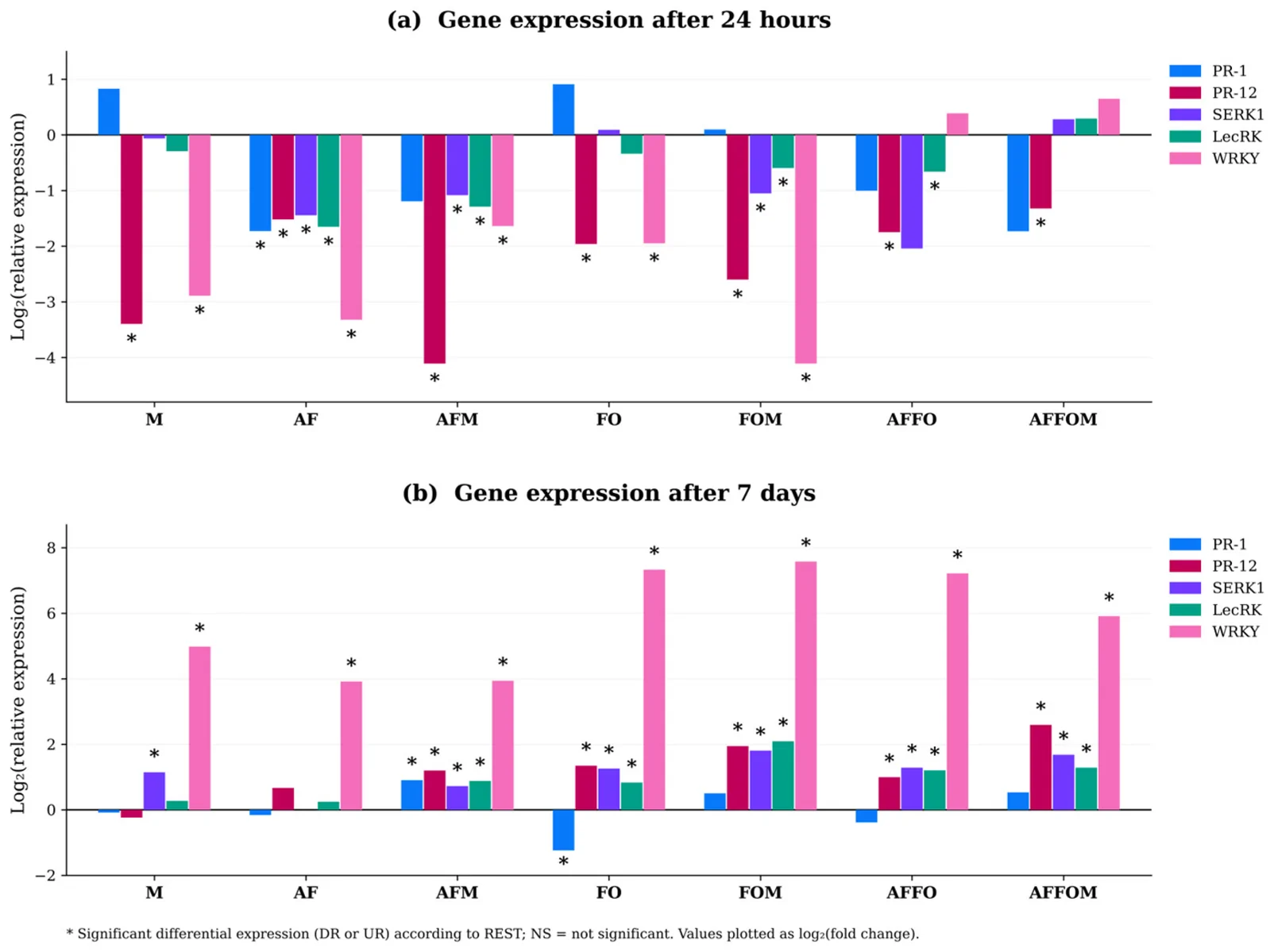
Relative expression of defense-related genes in *Passiflora edulis* plants inoculated with arbuscular mycorrhizal fungi (AMF) and challenged with *Fusarium oxysporum* f. sp. *passiflorae* (FoP) and/or fusaric acid (FA), evaluated at (**a**) 24 h and (**b**) 7 days after treatment. The relative expression levels of PePR-1, PePR-12, PeSERK1, PeLecRK, and PeWRKY were determined by RT-qPCR and analyzed using the Relative Expression Software Tool (REST 2009). Expression values are presented as log_2_-transformed fold changes relative to the control, with positive and negative values indicating upregulation and downregulation, respectively. Asterisks (*) indicate statistically significant differential expression (upregulation or downregulation) relative to the control, as determined by REST (*p* < 0.05). Treatments: M = AMF-inoculated plants; AF = plants treated with fusaric acid; AFM = AMF-inoculated plants treated with fusaric acid; FO = plants inoculated with FoP; FOM = AMF-inoculated plants inoculated with FoP; AFFO = plants treated with fusaric acid and inoculated with FoP; AFFOM = AMF-inoculated plants treated with fusaric acid and inoculated with FoP.

The expression profiles of the five defense-related genes differed markedly between 24 h and 7 days after treatment (Figure 2). At 24 h, the overall expression profile was predominantly characterized by transcriptional downregulation, with significant reductions in *PePR-12* and *PeWRKY* across several treatments. Significant downregulation of *PeSERK1* and *PeLecRK* was also observed in specific treatments, particularly AF, AFM, and FOM. In contrast, *PePR-1* showed a significant reduction only in the AF treatment. Notably, *PeWRKY* expression in the AFFO treatment was slightly increased (1.31-fold), although this change was not statistically significant. Overall, the early response was characterized by predominantly reduced transcript accumulation rather than broad transcriptional activation (Figure 2a; Table S1).

At 7 days, a markedly different expression profile emerged, characterized by broader transcriptional activation of the evaluated defense-related genes (Figure 2b). Significant upregulation of *PePR-12*, *PeSERK1*, and *PeLecRK* was detected in several treatments, particularly AFM and those involving FoP inoculation. In contrast, *PePR-1* showed significant upregulation only in AFM and significant downregulation in FO. Among the evaluated genes, *PeWRKY* exhibited the strongest and most consistent response, with significant upregulation detected in all treatments (Table S1).

The highest *PeWRKY* expression levels were observed in treatments involving FoP, particularly FO, FOM, and AFFO, reaching approximately 161-, 191-, and 149-fold relative to the control, respectively. *PeWRKY* was also the only evaluated gene showing significant differential expression in plants treated exclusively with FA at 7 days, reaching approximately 15-fold relative expression. Overall, these results revealed a pronounced temporal shift from predominantly reduced transcriptional responses at 24 h to broader activation of defense-related genes at 7 days, with *PeWRKY* exhibiting the most pronounced transcriptional response.

### 2.4. Relationships Between AMF Colonization and Defense-Related Gene Expression Differ Between Evaluation Times

**Figure 3:**
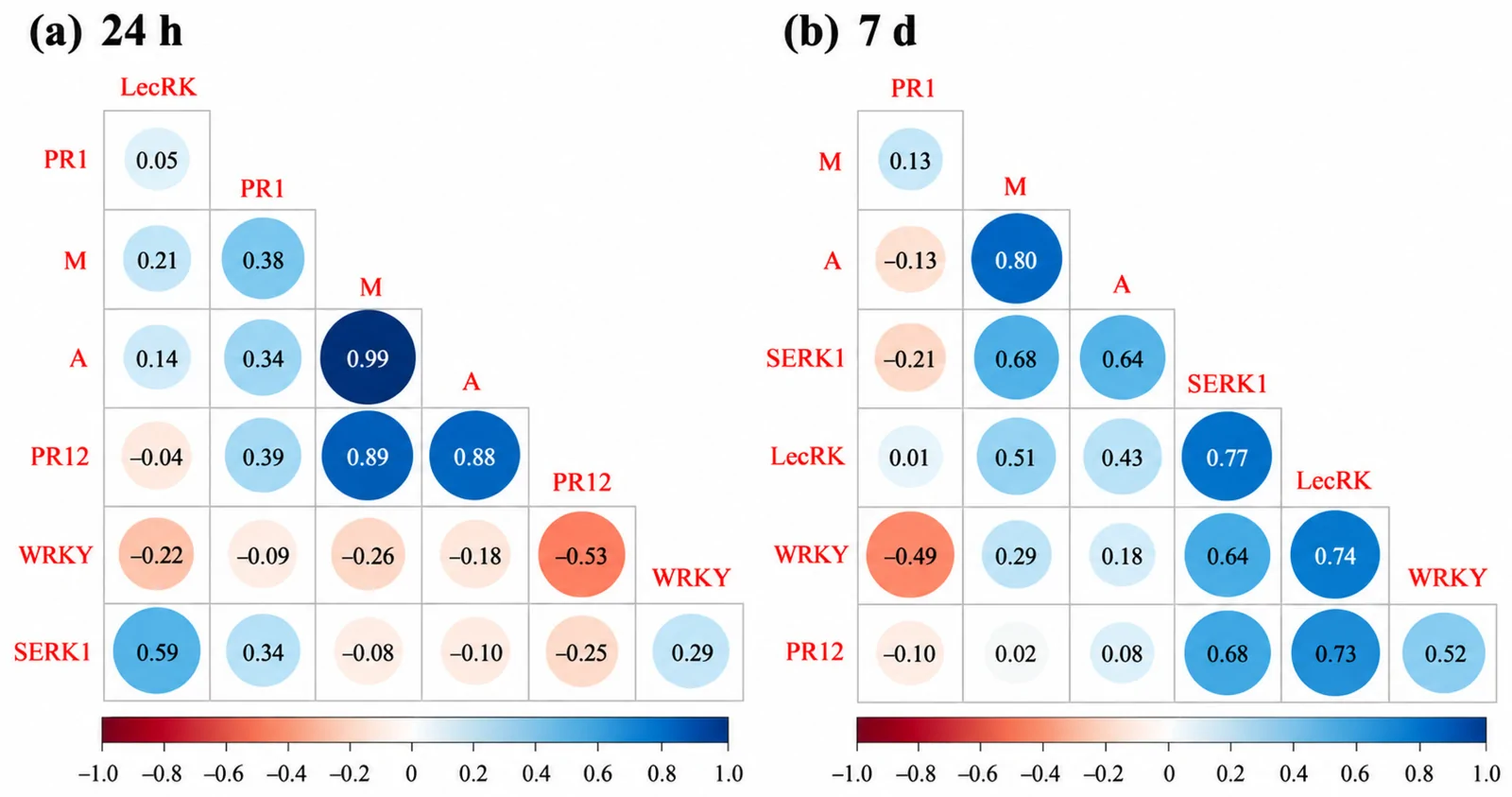
Correlation matrices between mycorrhizal colonization parameters and defense-related gene expression in *Passiflora edulis* at (**a**) 24 h and (**b**) 7 days after FoP inoculation and/or FA application. Correlations were calculated separately for each evaluation time. The variables included mycorrhizal intensity (M), arbuscular intensity (A), and the relative expression of *PePR-1*, *PePR-12*, *PeSERK1*, *PeLecRK*, and *PeWRKY*. Numbers within the circles represent correlation coefficients (*r*). Blue circles indicate positive correlations, and red circles indicate negative correlations, with circle size and color intensity proportional to the magnitude of the correlation. Values closer to +1 or −1 indicate stronger positive or negative correlations, respectively, whereas values closer to zero indicate weaker correlations.

Correlation analysis revealed distinct patterns of association between AMF colonization parameters and defense-related gene expression at 24 h and 7 days after FoP inoculation and/or FA application (Figure 3). At 24 h, mycorrhizal intensity (M) and arbuscular intensity (A) were strongly positively correlated (*r* = 0.99). Among the defense-related genes, *PePR-12* showed strong positive correlations with both mycorrhizal intensity (*r* = 0.89) and arbuscular intensity (*r* = 0.88). In contrast, *PeWRKY* was negatively correlated with mycorrhizal intensity (*r* = −0.26) and arbuscular intensity (*r* = −0.18), and a moderate negative correlation was observed between *PeWRKY* and *PePR-12* (*r* = −0.53). A moderate positive correlation was also observed between *PeSERK1* and *PePR-1* (*r* = 0.59) (Figure 3a).

At 7 days, the correlation pattern differed from that observed at 24 h (Figure 3b). Mycorrhizal intensity and arbuscular intensity remained strongly positively correlated (*r* = 0.80). *PeWRKY*, which was negatively correlated with mycorrhizal intensity at 24 h, showed a weak positive correlation with this parameter at 7 days (*r* = 0.29). *PeLecRK* was moderately positively correlated with mycorrhizal intensity (*r* = 0.51) and arbuscular intensity (*r* = 0.43), whereas *PePR-1* showed weak negative correlations with arbuscular intensity (*r* = −0.13) and *PeSERK1* (*r* = −0.21), and a moderate negative correlation with *PeWRKY* (*r* = −0.49).

Stronger positive associations among defense-related genes were evident at 7 days. *PeLecRK* was strongly positively correlated with *PeSERK1* (*r* = 0.77), *PeWRKY* (*r* = 0.74), and *PePR-12* (*r* = 0.73). Positive correlations were also observed between *PeSERK1* and *PePR-12* (*r* = 0.68), between *PeSERK1* and *PeWRKY* (*r* = 0.64), and between *PeWRKY* and *PePR-12* (*r* = 0.52). Overall, the two evaluation times displayed distinct correlation patterns, with stronger positive associations among several defense-related genes at 7 days.

## 3. Discussion

The present study provides evidence that prior colonization of *Passiflora edulis* by arbuscular mycorrhizal fungi (AMF) is associated with enhanced plant growth and temporally distinct defense-related responses following challenge with *Fusarium oxysporum* f. sp. *passiflorae* (FoP) and/or fusaric acid (FA). AMF-inoculated plants exhibited greater vegetative growth, whereas the transcriptional responses of the evaluated defense-related genes differed markedly between 24 h and 7 days. At 24 h, gene expression was predominantly reduced or limited, while at 7 days broader activation was observed, particularly for *PeWRKY*, *PeSERK1*, *PeLecRK*, and *PePR-12*. Together with the distinct correlation patterns observed at the two evaluation times, these findings suggest that AMF-associated defense in *P. edulis* involves temporal modulation of defense-related signaling rather than constitutive enhancement of defense-gene expression.

### 3.1. AMF Colonization Promotes Vegetative Growth and Establishes a Functional Symbiosis in P. edulis

The enhanced growth of AMF-inoculated *P. edulis* is consistent with the well-established contribution of arbuscular mycorrhizal symbiosis to plant nutrition and development. AMF extend the soil volume explored by the root system through their extraradical hyphal network and contribute substantially to nutrient acquisition, particularly phosphorus, although the magnitude of the resulting growth response depends on the plant–fungus combination and environmental conditions [19]. Smith and Smith [19], in particular, emphasize that nutrient transfer through the mycorrhizal pathway may be substantial even when growth responses vary among host–fungus combinations.

Positive effects of AMF have previously been demonstrated in *Passiflora*. Cavalcante et al. [20] showed that yellow passion fruit seedlings inoculated with *Gigaspora albida*, *G. margarita*, or *Glomus etunicatum* (=*Entrophospora etunicata*) exhibited greater growth than non-inoculated plants, with AMF inoculation increasing seedling vigor and potentially shortening the period required before field transplantation. Similarly, AMF inoculation promoted growth of *P. alata*, although the magnitude of the response depended on the fungal symbiont [21]. These previous findings are consistent with the present results, in which plant height was greater in AMF-inoculated plants from the first evaluation, whereas differences in leaf number and stem diameter became evident from 45 days onward. The original discussion likewise identified enhanced growth as one of the clearest effects of AMF inoculation in the present experiment.

The mixed inoculum of *G. albida* and *Entrophospora etunicata* used here therefore established a symbiotic association capable of promoting *P. edulis* growth before the plants were challenged with FoP and/or FA. This pre-existing difference in plant vigor is relevant when interpreting subsequent responses to biotic stress, because improved nutritional status and physiological performance represent important components of the overall benefits associated with AMF symbiosis [8,19].

Some fungal colonization was detected in treatments that had not been intentionally inoculated with AMF, indicating cross-contamination during the experiment. Consequently, mycorrhizal frequency and intensity in these treatments should be interpreted cautiously. Importantly, however, arbuscule-related variables more clearly differentiated AMF-inoculated from non-inoculated plants. Arbuscules are specialized intracellular structures involved in nutrient exchange between the fungal and plant partners and therefore provide strong evidence of an established functional symbiosis [6,19]. In the present study, arbuscular frequency exceeded 10% only in treatments previously inoculated with AMF, and neither FoP nor FA markedly disrupted arbuscule formation during the experimental period. The original observations similarly showed that arbuscule occurrence remained associated predominantly with the intentionally mycorrhized treatments despite the detected cross-contamination.

### 3.2. AMF-Associated Defense Responses Show a Pronounced Temporal Component

One of the most relevant findings of this study was the contrasting transcriptional response observed at 24 h and 7 days. Rather than producing uniformly elevated expression of defense-related genes, prior AMF colonization was associated with responses that differed according to the time elapsed after FoP inoculation and/or FA application. This behavior is consistent with the concept of mycorrhiza-induced resistance (MIR), in which AMF colonization alters the physiological and immune status of the host and may establish a primed state that modifies subsequent responses to biotic stress [8].

MIR should not necessarily be expected to result in constitutively elevated expression of defense genes. Instead, mycorrhizal plants may undergo adjustments in immune signaling during establishment of the symbiosis and subsequently exhibit altered timing or magnitude of defense responses following challenge [8]. Molecular communication between the plant and AMF involves extensive reprogramming of signaling and transcriptional pathways, allowing the host to accommodate the fungal symbiont while maintaining its capacity to respond to harmful microorganisms [6]. Thus, the predominantly reduced or limited expression observed at 24 h should not necessarily be interpreted as an absence of defense. It may instead represent an early regulatory phase during which signals derived from the established AMF symbiosis and those generated by FoP and/or FA are being integrated.

Evidence from other plant–AMF systems support this interpretation. In rice, AMF colonization modifies basal defense-related signaling and expression of defense genes [22] and can prime systemic responses that become more apparent following subsequent pathogen challenge [23]. Such responses illustrate the dynamic nature of AMF-mediated modulation of plant immunity and reinforce the importance of considering sampling time when evaluating molecular responses associated with MIR.

The broader activation observed at 7 days is consistent with this temporal perspective. At this time point, *PePR-12*, *PeSERK1*, *PeLecRK*, and particularly *PeWRKY* showed increased expression in several treatments. The transition from predominantly reduced or limited transcription at 24 h to broader activation at 7 days therefore supports a model in which the AMF-associated response of *P. edulis* involves changes in the kinetics and coordination of defense-related signaling rather than simple constitutive activation.

### 3.3. PeWRKY Is a Prominent Component of the Later Defense Response

Among the evaluated genes, *PeWRKY* exhibited the most pronounced response at 7 days and was the only gene showing significant expression changes in all treatments. Particularly high expression was observed in treatments involving FoP, supporting an important role for WRKY-mediated transcriptional regulation during the later response to the pathogen. WRKY transcription factors are major regulatory components of plant immunity and participate in networks connecting pathogen perception, phytohormone signaling, reactive oxygen species, and downstream defense responses [12].

The importance of WRKY transcription factors in *P. edulis* is also supported by genome-wide characterization of this gene family. Ma et al. [17] demonstrated that passion fruit contains multiple *PeWRKY* genes responsive to environmental stresses, emphasizing the involvement of this transcription factor family in stress-responsive regulatory networks. Although that study focused mainly on abiotic stress and flavonoid biosynthesis, it provides genomic evidence that WRKY-mediated regulation is an important component of stress responses in *P. edulis*.

The response to FA is particularly informative. *PeWRKY* was the only evaluated gene showing a significant expression change in plants exposed exclusively to FA at 7 days, reaching approximately 15-fold relative expression. FA is a non-host-specific phytotoxic metabolite produced by *F. oxysporum*, and genetic disruption of FA biosynthesis reduces fungal virulence, demonstrating that this metabolite contributes to the pathogenic process [4]. More recent transcriptomic and genetic evidence also indicates that FA participates in the molecular interaction between *F. oxysporum* and its host, affecting multiple defense- and stress-related pathways [5].

Nevertheless, the much greater *PeWRKY* expression observed in treatments containing FoP indicates that the response to the living pathogen cannot be explained solely by exposure to FA. Infection with FoP exposes the host to a broader range of fungal signals, potentially including microbe-associated molecular patterns, secreted molecules, tissue damage-derived signals, and metabolic perturbations. The stronger response to FoP-containing treatments is therefore compatible with *PeWRKY* acting as a regulatory node integrating multiple signals generated during the plant–pathogen interaction.

### 3.4. Receptor-Associated and Downstream Defense Genes Become More Coordinated at the Later Evaluation

The expression patterns of *PeSERK1* and *PeLecRK* provide further evidence for temporal organization of the defense response. SERK proteins are leucine-rich repeat receptor-like kinases that function as coreceptors in multiple receptor complexes involved in plant development and immunity [9,10]. LecRKs constitute another group of membrane-associated receptor-like kinases involved in extracellular signal perception and immune signaling [11]. The increased expression of *PeSERK1* and *PeLecRK* in several treatments at 7 days is therefore consistent with increased involvement of receptor-associated signaling during the later response to FoP and/or FA.

The correlation analysis reinforces this interpretation. At 7 days, *PeLecRK* was strongly positively correlated with *PeSERK1* (*r* = 0.77), *PeWRKY* (*r* = 0.74), and *PePR-12* (*r* = 0.73). *PeSERK1* was also positively correlated with *PePR-12* (*r* = 0.68) and *PeWRKY* (*r* = 0.64). Although these correlations do not establish direct regulatory interactions, they indicate that genes representing different components of the defense response (receptor-associated signaling, transcriptional regulation, and downstream defense) displayed more coordinated expression patterns at the later evaluation.

The behavior of *PePR-12* is particularly interesting in this context. PR-12 proteins comprise plant defensins, small cysteine-rich peptides with well-established antimicrobial properties, particularly against fungi [13,24]. At 24 h, *PePR-12* expression was strongly positively correlated with both mycorrhizal intensity (*r* = 0.89) and arbuscular intensity (*r* = 0.88). This close association between *PePR-12* and AMF colonization parameters suggests that the early behavior of this gene was closely related to the mycorrhizal status of the roots. The original analysis also identified the strong relationship between PR-12 expression and mycorrhizal colonization as one of the major early correlations.

By 7 days, however, *PePR-12* was strongly associated with *PeLecRK*, *PeSERK1*, and positively associated with *PeWRKY*. This difference suggests a transition from an early association with AMF colonization parameters toward integration with a broader set of defense-related genes. Such an interpretation is compatible with the antimicrobial role of plant defensins and with the broader activation of *PePR-12* observed at the later evaluation [13,24].

In contrast, *PePR-1* remained comparatively stable across most treatments. PR-1 is widely used as a molecular marker of plant defense, particularly responses associated with salicylic acid signaling, but PR families are not uniformly regulated, and their expression depends on the nature and timing of the stimulus [13]. The comparatively limited response of *PePR-1*, therefore, emphasizes that the defense response observed here did not involve generalized activation of all PR genes. Previous studies have also shown that AMF colonization can differentially modulate defense-related genes during establishment of the symbiosis and subsequent pathogen challenge [22,23].

### 3.5. Correlation Patterns Support Temporal Reorganization of Defense-Related Responses

The contrasting correlation patterns at 24 h and 7 days provide an additional perspective on the temporal organization of the response. At 24 h, the strongest relationships between AMF colonization and gene expression involved *PePR-12*, whereas *PeWRKY* was weakly negatively correlated with mycorrhizal intensity (*r* = −0.26) and moderately negatively correlated with *PePR-12* (*r* = −0.53). At 7 days, the association between *PeWRKY* and mycorrhizal intensity became weakly positive (*r* = 0.29), while stronger positive associations emerged among *PeSERK1*, *PeLecRK*, *PeWRKY*, and *PePR-12*. Importantly, these patterns represent correlations calculated independently at each evaluation time and should not be interpreted as statistical evidence that individual correlation coefficients changed significantly between 24 h and 7 days.

Comparable temporal modulation of defense-related signaling has been reported in other mycorrhizal systems. In grapevine colonized by *Rhizophagus irregularis*, Goddard et al. [25] found substantial changes in primary metabolism together with modifications in defense responses and signaling in both roots and leaves, including changes involving WRKY transcription factors and PR genes. Similarly, studies in rice have demonstrated that AMF symbiosis can alter basal defense-gene expression and prime plants for subsequent responses to pathogen infection [22,23]. These observations support the concept that AMF-associated defense is dynamic and that analyses based on a single sampling time may fail to capture important changes in the organization of the plant response.

Taken together, the expression and correlation data obtained here are consistent with temporal reprogramming of defense-related responses in *P. edulis*. At the early evaluation, transcriptional responses were predominantly reduced or limited, and the strongest associations with mycorrhizal colonization involved *PePR-12*. By 7 days, receptor-associated genes (*PeSERK1* and *PeLecRK*), the transcriptional regulator *PeWRKY*, and the downstream defense gene *PePR-12* showed broader activation and stronger positive associations with one another. This pattern is compatible with a transition toward a more coordinated defense-related state following challenge and suggests that prior AMF colonization may influence the kinetics and organization of the *P. edulis* response to FoP and FA.

However, these findings should not be interpreted as direct evidence that AMF increased resistance to fusariosis. The present study focused on plant growth, root colonization, and targeted gene-expression responses rather than disease severity, pathogen biomass, or plant survival. Moreover, correlations between colonization parameters and gene expression do not establish causal regulatory relationships. Interpretation is also constrained by the evaluation of a targeted set of five genes at only two time points and by the cross-contamination detected in some non-AMF treatments. Future studies incorporating additional sampling times, disease phenotyping, pathogen quantification, transcriptome-wide analyses, hormone profiling, and functional characterization of candidate genes would help determine whether the temporal molecular patterns identified here translate into increased resistance to FoP.

Overall, our findings indicate that prior colonization by *G. albida* and *E. etunicata* promotes the vegetative growth of *P. edulis* and is associated with temporally distinct defense-related responses following FoP and/or FA challenge. Rather than supporting a model of constitutively enhanced defense, the results point to a dynamic response characterized by limited early transcription followed by broader and more coordinated activation of receptor-associated, regulatory, and downstream defense genes. This temporal perspective provides a framework for understanding AMF-associated defense modulation in passion fruit and for future evaluation of AMF as a component of biologically based strategies for managing Fusarium wilt.

## 4. Materials and Methods

### 4.1. Experimental Site and Substrate

The experiment was conducted under greenhouse conditions at Embrapa Semiárido, Petrolina, Pernambuco, Brazil (9°04′37.7″ S, 40°18′57.1″ W). The mean temperature during the experimental period ranged from 25 to 29 °C, while relative humidity ranged from 55% to 75%. The soil used as substrate was classified as a Yellow Latosol and was collected from a preserved area of native Caatinga vegetation within the institution’s experimental field. Before use, the soil was autoclaved at 121 °C for three separate 1-h cycles and subsequently stored for 15 days. It had an electrical conductivity of 2.03 dS m^−1^, pH 7.4, available P of 101.35 mg kg^−1^, K of 0.72 cmolc dm^−3^, Na of 0.23 cmolc dm^−3^, and Ca of 4.00 cmolc dm^−3^. Particle-size distribution was 810 g kg^−1^ sand, 21 g kg^−1^ silt, and 168 g kg^−1^ clay, corresponding to a sandy loam texture. For plant cultivation, the autoclaved soil was mixed with vermiculite (1:1, *v/v*) and transferred to 4-L plastic pots.

### 4.2. Plant Material

Seeds of *Passiflora edulis* were obtained commercially and surface-disinfested in 0.05% (*v/v*) sodium hypochlorite for 10 min, rinsed with distilled water, and sown in plastic trays containing vermiculite. After seedling emergence and development of three fully expanded leaves, two seedlings were transplanted into each 4-L plastic pot containing autoclaved soil and vermiculite (1:1, *v/v*). Two seedlings were initially transplanted per pot to ensure successful plant establishment and survival. Following establishment, thinning was performed to retain only one plant per pot throughout the experiment.

### 4.3. AMF Inoculum Production and Plant Inoculation

Two arbuscular mycorrhizal fungi, *Gigaspora albida* (UNIVASF 05) and *Entrophospora etunicata* (UNIVASF 03), were obtained from the AMF inoculum collection of the Laboratory of Microbiology, Universidade Federal do Vale do São Francisco (UNIVASF), Campus Ciências Agrárias, Petrolina, Pernambuco, Brazil. The fungal isolates were propagated for three months under greenhouse conditions using maize (*Zea mays* L.) as the host plant in a substrate composed of washed sand and vermiculite (1:1, *v/v*).

At transplantation, the AMF inoculum was incorporated into the substrate in direct contact with the seedling root system. Each inoculated pot received approximately 400 glomerospores of *G. albida* and 400 glomerospores of *E. etunicata*, corresponding to a total of approximately 800 glomerospores per pot.

### 4.4. FoP Inoculum Production and Fusaric Acid Preparation

The isolate of *Fusarium oxysporum* f. sp. *passiflorae* (FoP) was obtained from the microorganism collection of Embrapa Mandioca e Fruticultura, Cruz das Almas, Bahia, Brazil. The isolate was cultured on potato dextrose agar (PDA) and incubated at 25 °C until sporulation. Conidia were harvested by gently scraping the colonies and suspending the resulting material in 10 mL of sterile distilled water. Conidial concentration was determined using a Neubauer hemocytometer and adjusted to 2 × 10^6^ conidia mL^−1^.

Fusaric acid (FA; Merck, Darmstadt, Germany) was prepared at a final concentration of 400 µM, based on a concentration previously reported to induce responses associated with fusariosis in *P. edulis* [26].

For FoP and FA application, 0.5 mL of the respective FoP conidial suspension or FA solution was infiltrated into the basal region of the stem using a syringe fitted with a needle. Control plants received 0.5 mL of sterile distilled water using the same procedure to account for potential effects of mechanical injury. Stem-base injection was selected to standardize the delivered dose of FoP and FA and to minimize variability associated with root penetration and initial infection establishment. This approach enabled a more controlled assessment of the temporal molecular responses to the pathogen and its phytotoxin. Although this method does not fully reproduce the natural root infection process of *F. oxysporum*, it provides a standardized experimental framework for comparing defense-related gene expression across treatments and evaluation times.

### 4.5. Experimental Design and Treatments

The experiment comprised 64 pots, each containing one *P. edulis* plant, with each plant representing an independent experimental unit. Thirty-two plants were inoculated with the AMF mixture (*G. albida* + *E. etunicata*), whereas 32 plants were maintained without intentional AMF inoculation. Vegetative growth was evaluated at 30, 45, and 70 days after AMF inoculation. For these variables, AMF-inoculated and non-inoculated plants were compared at each evaluation time.

After the final vegetative growth evaluation, plants were assigned to eight treatments combining AMF inoculation, FoP inoculation, and FA application (Table 2). The experiment was conducted in a completely randomized design with eight treatments and two evaluation times (24 h and 7 days after FoP inoculation and/or FA application), with four experimental replicates per treatment and evaluation time, totaling 64 experimental units.

The AMF treatment consisted of the mixture of *G. albida* and *E. etunicata* described above.

### 4.6. Vegetative Growth Measurements

Plant growth was evaluated based on plant height (cm), number of leaves, and stem diameter (cm). Measurements were performed before AMF inoculation and at 30, 45, and 70 days after inoculation. Plant height was measured using a ruler, and stem diameter was determined using a digital caliper.

For statistical analysis and presentation in Table 2, the measurements obtained at 30, 45, and 70 days were considered separately. This approach was adopted because the objective was to compare AMF-inoculated and non-inoculated plants within each evaluation time rather than to test temporal changes using a repeated-measures model.

### 4.7. Assessment of AMF Root Colonization

At the end of the experiment, subsamples of *P. edulis* roots were collected and stored in 50% ethanol until analysis. Roots were cleared and stained according to Dalpé and Séguin [27]. After staining, roots were cut into 30 fragments approximately 1 cm in length, and AMF colonization was evaluated according to Trouvelot et al. [18].

Four colonization parameters were determined: mycorrhizal frequency (F), mycorrhizal intensity (M), arbuscular frequency (a), and arbuscular intensity (A). F and M describe the occurrence and extent of overall mycorrhizal colonization, respectively, whereas a and A describe arbuscule occurrence and abundance.

### 4.8. RNA Extraction and cDNA Synthesis

Root tissues were collected at 24 h and 7 days after FoP inoculation and/or FA application. For gene expression analyses, three biological replicates were selected from the four available replicates per treatment and evaluation time based on RNA quality and integrity, as assessed by agarose gel electrophoresis and spectrophotometric measurements. Total RNA was extracted using the SV Total RNA Isolation System (Promega, Madison, WI, USA), including the DNase treatment step provided with the kit.

RNA integrity was evaluated by electrophoresis on 1% agarose gels stained with ethidium bromide, and RNA concentration and purity were assessed using a NanoDrop spectrophotometer (Thermo Fisher Scientific, Waltham, MA, USA). RNA samples were stored at −80 °C until cDNA synthesis.

First-strand cDNA was synthesized from 1 µg of total RNA using the GoScript Reverse Transcription Mix, Oligo(dT) (Promega, Madison, WI, USA), according to the manufacturer’s instructions, in a final reaction volume of 20 µL.

### 4.9. Selection of Target Genes and Primer Design

Five genes associated with signaling and defense responses in *P. edulis* were selected for RT-qPCR analysis: *PeWRKY*, encoding a WRKY transcription factor; *PeLecRK*, encoding an L-type lectin receptor-like kinase; *PeSERK1*, encoding somatic embryogenesis receptor-like kinase 1; and two pathogenesis-related genes, *PePR-1* and *PePR-12*, the latter encoding a plant defensin. Candidate sequences were identified in the *P. edulis* genome assembly ASM215610v1 available at NCBI.

Primers were designed using Primer3Plus [28], and primer specificity was evaluated using Primer-BLAST [29]. Primer design criteria included a GC content of 40–60%, expected amplicon length of 70–200 bp, and melting temperature of 57–60 °C. Potential primer-dimer formation was evaluated using PerlPrimer 1.1.21 [30].

Three candidate reference genes, *ADP*, *EF1α*, and *NDID*, were selected based on previous studies of reference-gene stability in *Passiflora* or related experimental systems. Their expression stability was evaluated using NormFinder [31] across the experimental treatments at 24 h and 7 days, both separately and in a combined analysis. Stability values for individual genes and the best two-gene combination are presented in Supplementary Table S2. All primers were synthesized and desalted by Exxtend Biotecnologia Ltda. (São Paulo, Brazil). Primer sequences, accession numbers, and expected amplicon sizes are presented in Table 3.

### 4.10. RT-qPCR and Relative Gene Expression Analysis

RT-qPCR assays were performed using a LineGene 9660 real-time PCR system, model FQD-96A (Bioer Technology, Hangzhou, China), with SYBR Green detection. Three biological replicates were analyzed per treatment and evaluation time, with three technical replicates for each biological sample.

Each reaction was performed in a final volume of 10 µL containing 5 µL of GoTaq qPCR Master Mix 2× (Promega, Madison, WI, USA), 1 µL of cDNA, 0.3 µL of each primer at 5 µM, and nuclease-free water to the final volume. The thermal cycling program consisted of an initial denaturation at 95 °C for 2 min, followed by 40 cycles at 95 °C for 15 s and 60 °C for 1 min. Melting-curve analysis was performed from 65 to 95 °C after amplification to verify the specificity of the PCR products. No-template controls (NTCs) were included in all assays. Representative amplification curves, melting-curve profiles, and standard curves for the five target genes and three reference genes are provided in Supplementary Figures S1–S3, respectively, to document amplification performance, specificity, and efficiency.

Amplification efficiency was estimated from standard curves using the equation *E* = 10^(−1/slope)^. Relative gene expression was analyzed using the Relative Expression Software Tool (REST 2009) [34], considering PCR amplification efficiencies and differences in crossing-point values between treatment and control samples. Three reference genes (ADP, EF1α, and NDID) were jointly used for normalization of the expression data. Statistical significance of expression ratios was assessed using the Pair-Wise Fixed Reallocation Randomization Test implemented in REST [34].

### 4.11. Statistical Analysis

Vegetative growth data were tested for normality of residuals and homogeneity of variances before analysis. Plant height, number of leaves, and stem diameter were analyzed independently at 30, 45, and 70 days after AMF inoculation. At each evaluation time, AMF-inoculated and non-inoculated plants were compared by analysis of variance (ANOVA), and means were compared using Tukey’s test at *p* < 0.05.

AMF colonization variables were analyzed among the eight experimental treatments, and means were compared using Tukey’s test at *p* < 0.05. Statistical analyses were performed using R software (version 4.2.3; R Foundation for Statistical Computing, Vienna, Austria) [35]. The R packages RAMF [36], ExpDes.pt [37], and ggplot2 [38] were used for analysis and/or graphical representation, as appropriate.

Relationships between mycorrhizal intensity (M), arbuscular intensity (A), and relative expression of *PePR-1*, *PePR-12*, *PeSERK1*, *PeLecRK*, and *PeWRKY* were evaluated separately at 24 h and 7 days. Correlation matrices were generated using the corrplot package [39].

Relative gene-expression data were analyzed separately using REST as described above and were therefore not subjected to the ANOVA/Tukey procedure used for the vegetative growth and colonization variables.

## 5. Conclusions

The present study demonstrates that inoculation with the arbuscular mycorrhizal fungi (AMF) *Gigaspora albida* and *Entrophospora etunicata* promotes vegetative growth in *Passiflora edulis* and is associated with temporally distinct defense-related transcriptional responses following exposure to *Fusarium oxysporum* f. sp. *passiflorae* (FoP) and/or fusaric acid (FA). Gene expression was predominantly reduced or limited at 24 h, whereas broader activation was observed at 7 days, particularly for *PeWRKY*, *PeSERK1*, *PeLecRK*, and *PePR-12*. The pronounced induction of *PeWRKY* in FoP-containing treatments highlights its potential involvement in the later defense response. Furthermore, the distinct correlation patterns observed at the two evaluation times suggest a temporal shift in the relationships between mycorrhizal colonization and defense-related gene expression, with greater coordination among receptor-associated, regulatory, and downstream defense genes at 7 days. These findings are consistent with AMF-associated modulation of plant defense signaling and provide insights into the molecular responses of *P. edulis* to FoP and FA. However, whether these transcriptional changes translate into enhanced resistance to Fusarium wilt remains to be established through direct disease assessments. Overall, this study provides a foundation for further investigations into the potential application of AMF in sustainable strategies for managing Fusarium wilt in passion fruit.

## Figures and Tables

**Table 1:** Effect of AMF inoculation on vegetative growth of *Passiflora edulis* seedlings at 30, 45, and 70 days after inoculation.

Growth Trait	30 Days		45 Days		70 Days	
	Non-AMF	AMF	Non-AMF	AMF	Non-AMF	AMF
Plant height (cm)	11.69 b	15.47 a	19.49 b	43.33 a	61.12 b	89.92 a
Number of leaves	7.12 a	7.12 a	10.05 b	13.08 a	12.47 b	14.16 a
Stem diameter (cm)	0.10 a	0.10 a	0.14 b	0.41 a	0.48 b	0.59 a

Note: AMF, arbuscular mycorrhizal fungi. AMF-inoculated plants received a mixture of *Entrophospora etunicata* and *Gigaspora albida*. Each evaluation time was analyzed independently. Within each time point and growth trait, means followed by the same lowercase letter do not differ significantly according to Tukey’s test at *p* < 0.05.

**Table 2:** Experimental treatments applied to *Passiflora edulis* plants according to prior AMF inoculation and subsequent challenge with *Fusarium oxysporum* f. sp. *passiflorae* (FoP) and/or fusaric acid (FA).

Treatment	Description
C	Non-AMF-inoculated plants without FoP or FA
M	AMF-inoculated plants without FoP or FA
AF	Non-AMF-inoculated plants treated with FA
AFM	AMF-inoculated plants treated with FA
FO	Non-AMF-inoculated plants inoculated with FoP
FOM	AMF-inoculated plants inoculated with FoP
AFFO	Non-AMF-inoculated plants treated with FA and inoculated with FoP
AFFOM	AMF-inoculated plants treated with FA and inoculated with FoP

**Table 3:** Primers used for RT-qPCR analysis of defense-related and reference genes in *Passiflora edulis*.

Gene	Accession Number	Primer Sequence (5′–3′)	Amplicon (bp)	Reference
*PeLecRK*	MUZT01080488.1	F: AGTCGGCTCTATTGGCCTTC; R: ACCGGGAAGGCTACAACAAG	90	This study
*PeSERK1*	KT373980.1	F: GGACCGGCCTAAGATGTCAG; R: CACCTTTTGCCACTCATCCC	82	This study
*PeWRKY*	MUZT01152844.1	F: AGGAAGTGAGAACCCACGTAG; R: TTTGTCCATCCGGAGACCTC	89	This study
*PePR-1*	MUZT01001854.1	F: TGGGTTGCATTGGGGAGTC; R: TCTTTGACCAGCCCACCATC	128	This study
*PePR-12*	MUZT01199879.1	F: AGGACATGCGAGTCACAGAG; R: CACTTGCCACCGATGAAACC	101	This study
*EF1α*	DQ447160.1	F: GTTAAGGATTTGAAGCGTGG; R: ATGTGTGATGTGTGGCAGT	155	Munhoz [32]
*NDID*	AB304270.1	F: GTCGTCACTCTCTCTTTACG; R: TCATTTCATCACCGTCCATC	172	Munhoz [32]
*ADP*	XM_015716874.2	F: GAGAGATGCTGTGCTGCTTG; R: TGAAGGCCGAGCTTATCAGT	90	Cassol et al. [33]

## Data Availability

The original contributions presented in this study are included in the article/Supplementary Materials. Further inquiries can be directed to the corresponding authors.

## Supplementary Materials

The following supporting information can be downloaded at https://www.mdpi.com/article/10.3390/ijms27219495/s1.

## Abbreviations

The following abbreviations are used in this manuscript:

**Abbreviation**	**Definition**
A	Arbuscular intensity
a	Arbuscular frequency
AF	Plants treated with fusaric acid
AFFO	Plants treated with fusaric acid and inoculated with FoP
AFFOM	AMF-inoculated plants treated with fusaric acid and inoculated with FoP
AFM	AMF-inoculated plants treated with fusaric acid
AMF	Arbuscular mycorrhizal fungi
ANOVA	Analysis of variance
C	Non-inoculated control
cDNA	Complementary DNA
DNA	Deoxyribonucleic acid
F	Mycorrhizal frequency
FA	Fusaric acid
FO	Plants inoculated with FoP
FOM	AMF-inoculated plants inoculated with FoP
FoP	*Fusarium oxysporum* f. sp. *passiflorae*
M	Mycorrhizal intensity (colonization parameter) or AMF-inoculated plants (treatment code)
MIR	Mycorrhiza-induced resistance
NCBI	National Center for Biotechnology Information
NTC	No-template control
PDA	Potato dextrose agar
REST	Relative Expression Software Tool
RNA	Ribonucleic acid
RT-qPCR	Reverse transcription quantitative polymerase chain reaction
