# Supplementary material for: Arbuscular Mycorrhizal Fungi Modulate the Temporal Defense Response of Passiflora edulis to Fusarium oxysporum
Source: Int J Mol Sci. 2026 Oct 24;27(21):9495. doi: 10.3390/ijms27219495 (PMC13763165; doi:10.3390/ijms27219495)

Figure S1. Representative amplification curves obtained by reverse transcription quantitative PCR (RT-qPCR) for five defense-related target genes (*PePR-1*, *PePR-12*, *PeSERK1*, *PeLecRK*, and *PeWRKY*) and three reference genes (*ADP*, *EF1α*, and *NDID*) in *Passiflora edulis*. Fluorescence intensity is plotted against amplification cycle number.

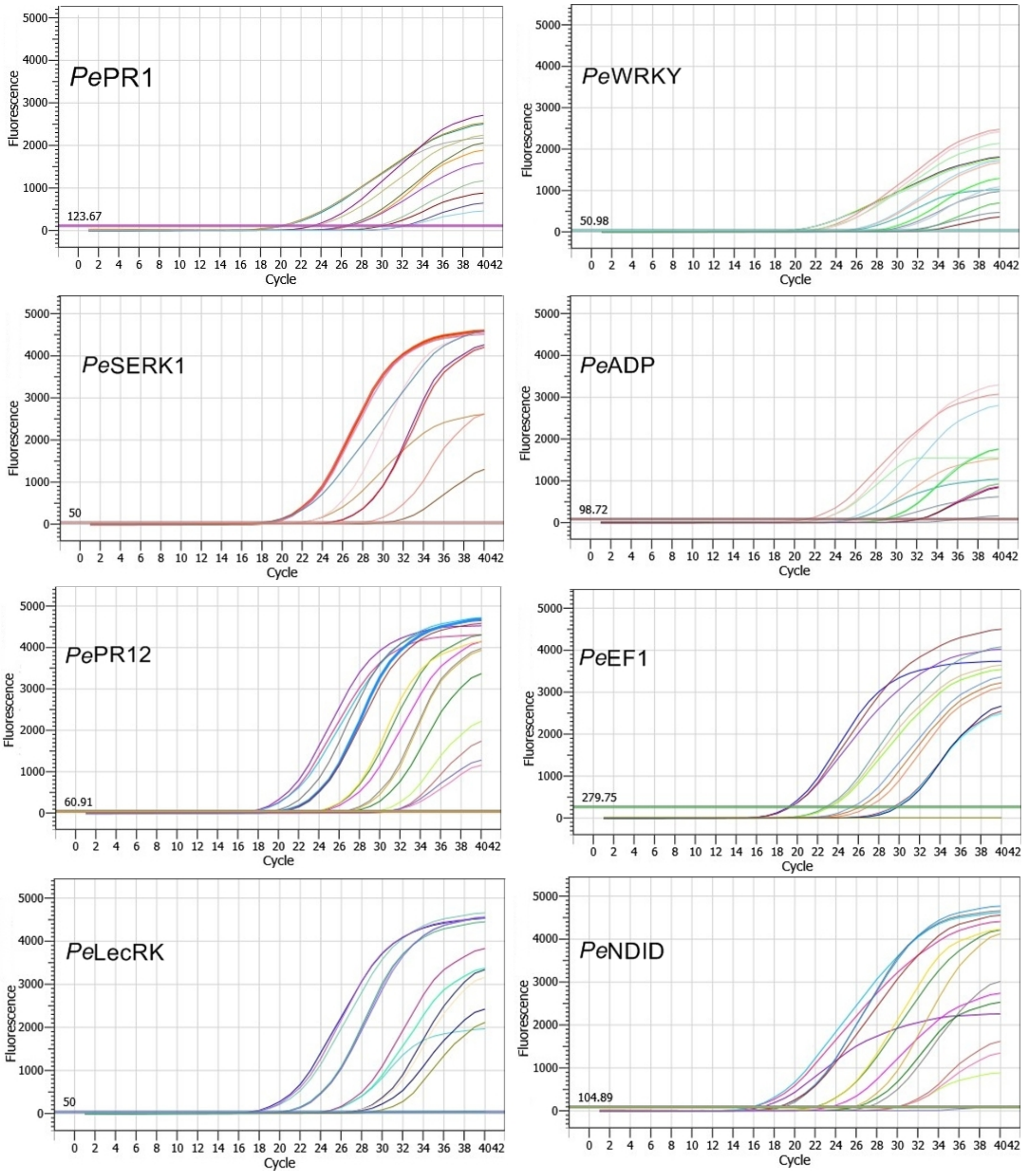

Figure S2. Melting-curve profiles obtained from RT-qPCR assays for five defense-related target genes (*PePR-1*, *PePR-12*, *PeSERK1*, *PeLecRK*, and *PeWRKY*) and three reference genes (*ADP*, *EF1α*, and *NDID*) in *Passiflora edulis*. Melting-curve analysis was performed to assess amplification specificity and detect potential nonspecific products.

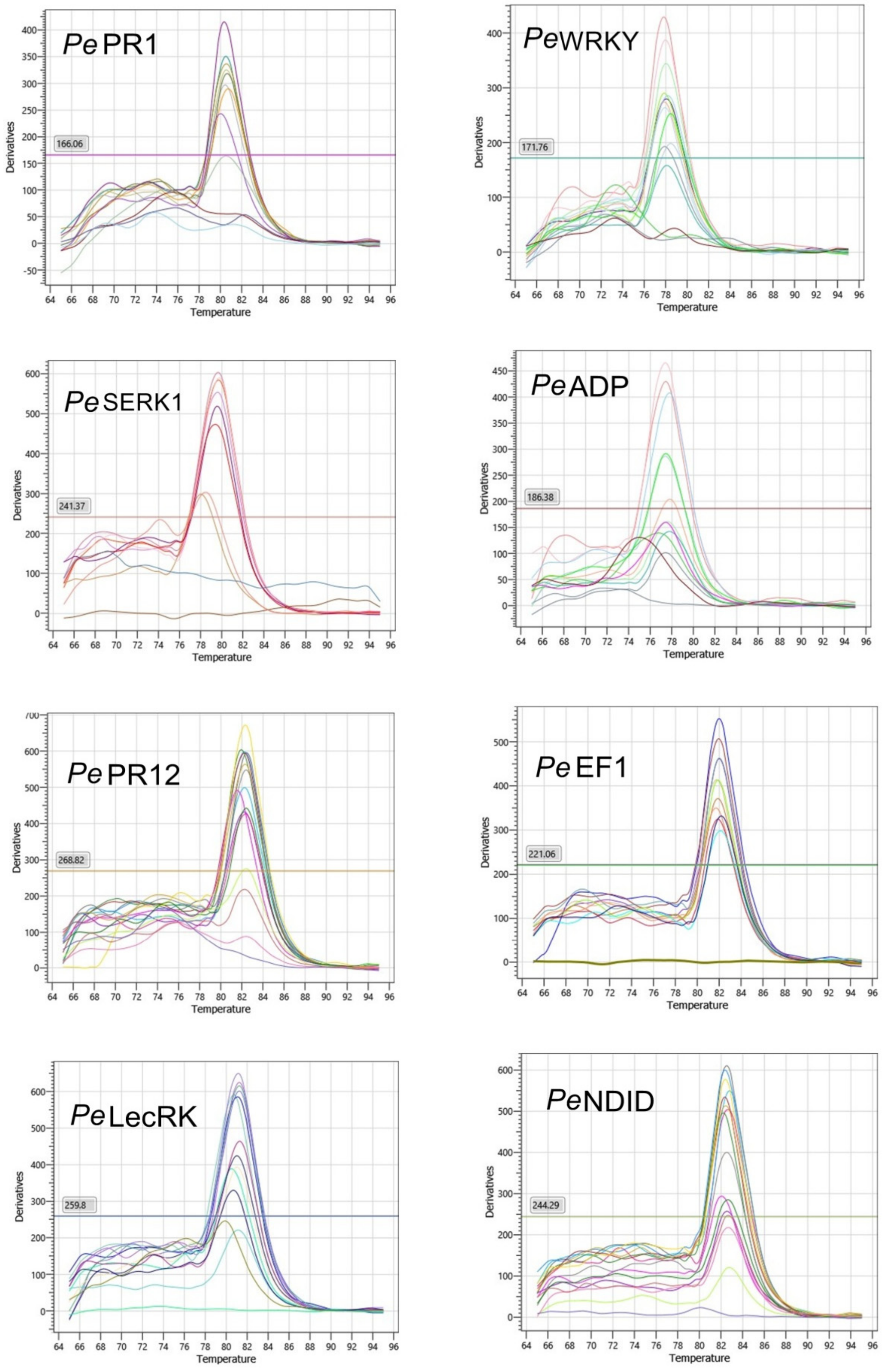

Figure S3. Standard curves generated for five defense-related target genes (*PePR-1*, *PePR-12*, *PeSERK1*, *PeLecRK*, and *PeWRKY*) and three reference genes (*ADP*, *EF1α*, and *NDID*) in *Passiflora edulis*. The curves illustrate the relationship between quantification cycle (Cq) values and template concentration and were used to estimate amplification efficiency. Regression parameters, including slope, y-intercept, and correlation coefficient, are presented for each gene.

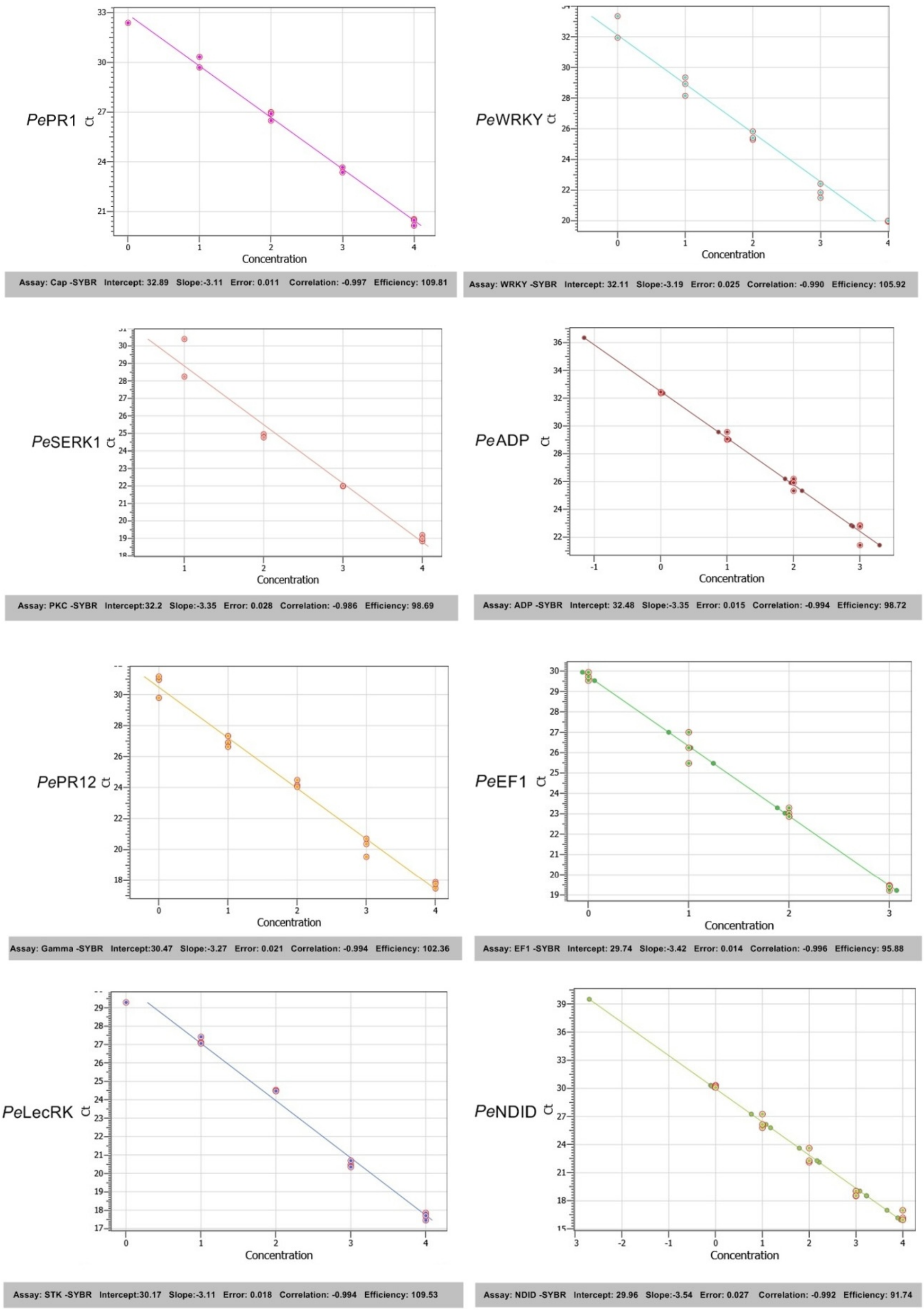

Supplement: Supplementary file 1 [file ijms-27-09495-s001.zip › ijms-4648917-supplementary/Supplementary_Figures_S1-S3.pdf]
