# Supplementary material for: Arbuscular Mycorrhizal Fungi Modulate the Temporal Defense Response of Passiflora edulis to Fusarium oxysporum
Source: Int J Mol Sci. 2026 Oct 24;27(21):9495. doi: 10.3390/ijms27219495 (PMC13763165; doi:10.3390/ijms27219495)
Supplement: Supplementary file 1 [file ijms-27-09495-s001.zip › ijms-4648917-supplementary/Tables_S1_Expression relative REST_and_S2_NormFinder stability values of candidate reference genes.pdf]

Table S1. Relative expression of defense-related genes in *Passiflora edulis* at 24 h and 7 days after treatment, as determined by the Relative Expression Software Tool (REST).

		**M 24h**		
**Gene**	**Relative Expression**	**SE**	**95% CI**	**P(H1)**
PR-1	1.777 (NS)	0.367 - 8.153	0.085 - 16.363	0.291
PR-12	**0.095 (DR)**	0.025 - 0.368	0.009 - 0.535	0.000
SERK1	0.957 (NS)	0.351 - 3.021	0.112 - 4.377	0.901
LectinaRK	0.816 (NS)	0.454 - 1.510	0.301 - 2.277	0.308
WRKY	**0.135 (DR)**	0.028 - 0.510	0.008 - 0.984	0.000
		**AF 24h**		
**Gene**	**Relative Expression**	**SE**	**95% CI**	**P(H1)**
PR-1	**0.302 (DR)**	0.067 - 1.317	0.021 - 3.466	0.042
PR-12	**0.349 (DR)**	0.165 - 0.750	0.114 - 1.553	0.002
SERK1	**0.367 (DR)**	0.112 - 0.919	0.056 - 1.435	0.010
LectinaRK	**0.318 (DR)**	0.133 - 0.744	0.063 - 1.161	0.001
WRKY	**0.100 (DR)**	0.039 - 0.263	0.013 - 0.463	0.000
		**AFM 24h**		
**Gene**	**Relative Expression**	**SE**	**95% CI**	**P(H1)**
PR-1	0.437 (NS)	0.066 - 3.133	0.013 - 9.573	0.230
PR-12	**0.058 (DR)**	0.019 - 0.164	0.011 - 0.524	0.000
SERK1	**0.472 (DR)**	0.179 - 1.048	0.073 - 2.820	0.035
LectinaRK	**0.409 (DR)**	0.170 - 0.949	0.088 - 2.443	0.012
WRKY	**0.321 (DR)**	0.128 - 0.676	0.075 - 1.645	0.002
		**FO 24h**		
**Gene**	**Relative Expression**	**SE**	**95% CI**	**P(H1)**
PR-1	1.877 (NS)	0.411 - 7.795	0.102 - 15.582	0.217
PR-12	**0.257 (DR)**	0.080 - 0.761	0.055 - 1.912	0.004
SERK1	1.065 (NS)	0.390 - 2.900	0.180 - 4.138	0.847
LectinaRK	0.790 (NS)	0.386 - 1.404	0.215 - 2.290	0.300
WRKY	**0.259 (DR)**	0.085 - 0.786	0.033 - 1.463	0.002
		**FOM 24h**		
**Gene**	**Relative Expression**	**SE**	**95% CI**	**P(H1)**
PR-1	1.070 (NS)	0.242 - 4.501	0.058 - 10.135	0.891
PR-12	**0.165 (DR)**	0.081 - 0.410	0.040 - 0.683	0.000
SERK1	**0.482 (DR)**	0.184 - 1.149	0.077 - 1.729	0.022
LectinaRK	**0.662 (DR)**	0.397 - 1.068	0.278 - 1.450	0.024
WRKY	**0.058 (DR)**	0.014 - 0.387	0.006 - 2.159	0.000
		**AFFO 24h**		
**Gene**	**Relative Expression**	**SE**	**95% CI**	**P(H1)**
PR-1	0.499 (NS)	0.098 - 1.787	0.029 - 4.971	0.149
PR-12	**0.298 (DR)**	0.133 - 0.686	0.080 - 1.262	0.000
SERK1	0.243 (NS)	0.155 - 1.645	0.075 - 2.311	0.290
LectinaRK	**0.633 (DR)**	0.369 - 1.108	0.209 - 1.808	0.028
WRKY	1.307 (NS)	0.539 - 2.707	0.330 - 4.661	0.110
		**AFFOM 24h**		
**Gene**	**Relative Expression**	**SE**	**95% CI**	**P(H1)**

PR-1	0.301 (NS)	0.023 - 5.038	0.000 - 13.668	0.373
PR-12	**0.400 (DR)**	0.156 - 1.237	0.048 - 1.981	0.017
SERK1	1.215 (NS)	0.467 - 3.040	0.161 - 4.973	0.564
LectinaRK	1.226 (NS)	0.483 - 2.978	0.247 - 5.635	0.525
WRKY	1.566 (NS)	0.595 - 4.225	0.146 - 8.481	0.230
**M 7D**				
**Gene**	**Relative Expression**	**SE**	**95% CI**	**P(H1)**
PR-1	0.945 (NS)	0.338 - 2.674	0.074 - 5.633	0.911
PR-12	0.848 (NS)	0.264 - 2.476	0.129 - 4.406	0.647
SERK1	**2.209 (UR)**	1.347 - 3.225	0.774 - 7.808	0.001
LectinaRK	1.208 (NS)	0.516 - 2.696	0.293 - 4.710	0.479
WRKY	**31.581(UR)**	12.365 - 83.481	7.278 - 186.021	0.000
**AF 7D**				
**Gene**	**Relative Expression**	**SE**	**95% CI**	**P(H1)**
PR-1	0.897 (NS)	0.421 - 1.831	0.246 - 4.059	0.691
PR-12	1.589 (NS)	0.581 - 3.827	0.349 - 10.387	0.164
SERK1	1.001 (NS)	0.595 - 1.680	0.432 - 3.041	0.998
LectinaRK	1.187 (NS)	0.608 - 2.521	0.383 - 3.284	0.425
WRKY	**15.084 (UR)**	6.033 - 37.852	2.762 - 66.623	0.000
**AFM 7D**				
**Gene**	**Relative Expression**	**SE**	**95% CI**	**P(H1)**
PR-1	**1.867 (UR)**	0.851 - 4.308	0.493 - 9.753	0.041
PR-12	**2.293 (UR)**	1.136 - 5.447	0.631 - 8.876	0.005
SERK1	**1.651 (UR)**	0.841 - 3.102	0.639 - 6.728	0.037
LectinaRK	**1.841 (UR)**	1.005 - 3.591	0.661 - 5.290	0.014
WRKY	**15.354 (UR)**	5.466 - 61.412	2.718 - 129.610	0.000
**FO 7D**				
**Gene**	**Relative Expression**	**SE**	**95% CI**	**P(H1)**
PR-1	**0.424 (DR)**	0.199 - 0.874	0.124 - 1.869	0.008
PR-12	**2.545 (UR)**	1.302 - 5.750	0.568 - 11.394	0.004
SERK1	**2.392 (UR)**	1.435 - 4.195	0.785 - 8.342	0.001
LectinaRK	**1.782 (UR)**	0.855 - 3.929	0.534 - 7.919	0.036
WRKY	**160.887 (UR)**	53.936 - 458.575	23.085 - 705.217	0.000
**FOM 7D**				
**Gene**	**Relative Expression**	**SE**	**95% CI**	**P(H1)**
PR-1	1.416 (NS)	0.373 - 8.462	0.202 - 24.438	0.504
PR-12	**3.853 (UR)**	1.008 - 15.852	0.585 - 122.435	0.005
SERK1	**3.506 (UR)**	1.151 - 15.260	0.700 - 32.695	0.004
LectinaRK	**4.262 (UR)**	1.206 - 20.692	0.694 - 98.642	0.002
WRKY	**191.118 (UR)**	57.789 - 973.036	28.924 - 2,771.737	0.000
**AFFO 7D**				
**Gene**	**Relative Expression**	**SE**	**95% CI**	**P(H1)**
PR-1	0.767 (NS)	0.295 - 1.774	0.120 - 5.082	0.478
PR-12	**1.997 (UR)**	0.879 - 4.731	0.448 - 8.656	0.032
SERK1	**2.444 (UR)**	0.932 - 7.767	0.471 - 17.608	0.026
LectinaRK	**2.306 (UR)**	0.869 - 5.815	0.404 - 15.052	0.026

WRKY	**148.640 (UR)**	46.995 - 478.843	22.004 - 735.152	0.000
**AFFOM 7D**				
**Gene**	**Relative Expression**	**SE**	**95% CI**	**P(H1)**
PR-1	1.447 (NS)	0.486 - 3.445	0.262 - 8.278	0.270
PR-12	**6.014 (UR)**	1.341 - 34.563	0.463 - 98.764	0.005
SERK1	**3.206 (UR)**	1.750 - 6.946	1.008 - 10.734	0.000
LectinaRK	**2.444 (UR)**	1.256 - 4.871	0.751 - 10.933	0.001
WRKY	**60.241 (UR)**	17.727 - 300.606	8.591 - 509.479	0.000

Note: Relative expression values are presented as fold changes relative to the control. DR = significantly downregulated; UR = significantly upregulated; NS = not significant. Statistical significance was determined using the Relative Expression Software Tool (REST; $p < 0.05$). Treatments: M = plants inoculated with arbuscular mycorrhizal fungi (AMF); AF = plants treated with fusaric acid (FA); AFM = AMF-inoculated plants treated with FA; FO = plants inoculated with *Fusarium oxysporum* f. sp. *passiflorae* (FoP); FOM = AMF-inoculated plants inoculated with FoP; AFFO = plants treated with FA and inoculated with FoP; AFFOM = AMF-inoculated plants treated with FA and inoculated with FoP. Sampling times: 24 h and 7 days after treatment.

Table S2. NormFinder stability values of candidate reference genes in *Passiflora edulis* across experimental treatments at 24 h, 7 days, and in the combined analysis.

**Reference gene(s)**	**24 h**	**7 days**	**Combined analysis**
ADP	0.1551	0.2473	0.2121
EF1α	0.1829	0.3552	0.2661
NDID	0.2294	0.3251	0.2913
Best two-gene combination: ADP + EF1α	0.1293	0.2279	0.2033

Table note: Lower NormFinder stability values indicate greater expression stability. ADP was the most stable individual reference gene in all three analyses, whereas ADP and EF1α constituted the best two-gene combination. All three reference genes (ADP, EF1α, and NDID) were used for normalization of RT-qPCR expression data in REST. The combined analysis included samples from both evaluation times.
